# Identification of Interactions between Proteins Encoded by Grapevine Leafroll-Associated Virus 3

**DOI:** 10.3390/v15010208

**Published:** 2023-01-11

**Authors:** Ilani Mostert, Rachelle Bester, Johan T. Burger, Hans J. Maree

**Affiliations:** 1 Department of Genetics, Stellenbosch University, Private Bag X1, Matieland 7602, South Africa; 2 Citrus Research International, P.O. Box 2201, Matieland 7602, South Africa

**Keywords:** *Closteroviridae*, *Ampelovirus*, protein-protein interactions, yeast two-hybrid, bimolecular fluorescence complementation, virion assembly

## Abstract

The roles of proteins encoded by members of the genus *Ampelovirus*, family *Closteroviridae* are largely inferred by sequence homology or analogy to similarly located ORFs in related viruses. This study employed yeast two-hybrid and bimolecular fluorescence complementation assays to investigate interactions between proteins of grapevine leafroll-associated virus 3 (GLRaV-3). The p5 movement protein, HSP70 homolog, coat protein, and p20B of GLRaV-3 were all found to self-interact, however, the mechanism by which p5 interacts remains unknown due to the absence of a cysteine residue crucial for the dimerisation of the closterovirus homolog of this protein. Although HSP70h forms part of the virion head of closteroviruses, in GLRaV-3, it interacts with the coat protein that makes up the body of the virion. Silencing suppressor p20B has been shown to interact with HSP70h, as well as the major coat protein and the minor coat protein. The results of this study suggest that the virion assembly of a member of the genus *Ampelovirus* occurs in a similar but not identical manner to those of other genera in the family *Closteroviridae*. Identification of interactions of p20B with virus structural proteins provides an avenue for future research to explore the mechanisms behind the suppression of host silencing and suggests possible involvement in other aspects of the viral replication cycle.

## 1. Introduction

**Figure 1:**
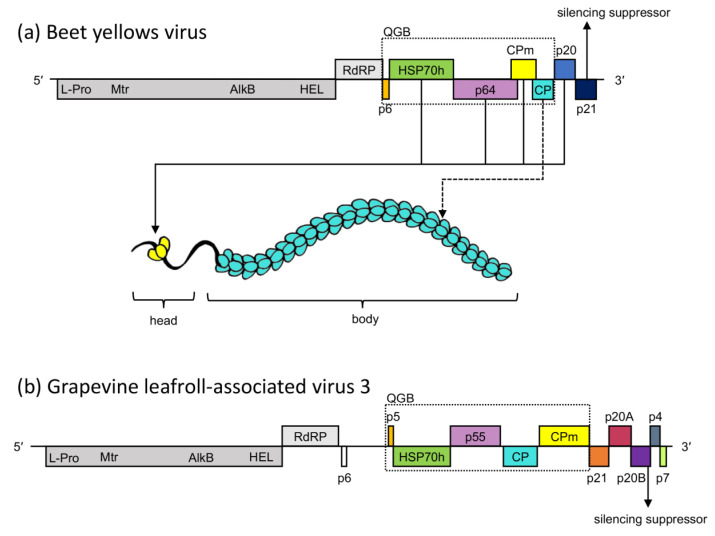
(**a**) Genome organisation of beet yellows virus (BYV) from the genus *Closterovirus*, family *Closteroviridae*, with an illustration of the bipartite virion of members of this family. Involvement of the structural proteins in the two parts of the virion is indicated by arrows. (**b**) Genome organisation of grapevine leafroll-associated virus 3 (GLRaV-3). Genes encoding homologous proteins are filled with the same colour. L-Pro: leader papain-like protease; Mtr: methyltransferase; AlkB: AlkB domain; HEL: helicase; RdRP: RNA dependent RNA polymerase; HSP70h: heat shock protein 70 homolog, CP: coat protein, CPm: minor coat protein; QGB: quintuple gene block.

Knowledge of the molecular architecture of virus particles, how they are assembled and disassembled, and how they move between cells is essential to understand the way these viruses function within a host. The family *Closteroviridae* contains seven genera, of which four, *Closterovirus*, *Ampelovirus*, *Velarivirus,* and *Crinivirus*, were established prior to 2021 and studied more extensively [1,2]. Members of this family possess positive-sense RNA genomes encapsidated in virions with a bipolar architecture made up of two distinct but related coat proteins (Figure 1a) [3,4].

The proteins required for virion assembly and movement of beet yellows virus (BYV) and citrus tristeza virus (CTV), both belonging to the genus *Closterovirus*, have been extensively studied. The main body of BYV covers 95% of the genome from the 3′ end and consists of subunits of the major coat protein (CP), p22, arranged in a helical pattern [5]. The remainder of the genomic RNA is encapsidated by p24, the minor coat protein (CPm), and is commonly referred to as the ‘head’ of the virion [5,6]. In addition to p24, three other proteins are incorporated into the virion head structure (Figure 1a). The first, HSP70h, is a homolog of the cellular family of ~70 kDa heat shock proteins (HSP70s) and is required for proper head formation [7]. A second protein, p64, is embedded into the virion head by its C-terminus [8], possesses RNA-binding properties [9], and its CTV-encoded homolog has been proposed to bind near the 5′ end of the genome to prevent encapsidation of the whole virion by CPm [10]. All four of these structural proteins (heat shock protein 70 homolog (HSP70h), p64, CPm, and CP) are necessary for cell-to-cell movement of virions [7,11,12]. BYV p20 was shown to form part of the virion head structure. Although p20 is not required for virion assembly or cell-to-cell movement, it is essential for long-distance movement of the virus in its host [13]. No homologs of p20 have been found in other members of the family *Closteroviridae*. However, proteins encoded by genes in a similar location in the genomes of related viruses have been implicated in systemic spread [14] and the suppression of host RNA interference defence mechanisms [15,16,17]. One such protein, GLRaV-3 p20B, has been shown to supress RNA silencing in *N. benthamiana* (Figure 1b) [18]. Along with the structural proteins, a small membrane-associated protein, p6, is required for cell-to-cell movement of BYV [13,19]. Collectively, homologs of BYV p6, HSP70h, CP, CPm, and p64, make up the quintuple gene block (QGB) encoded by all members of the family *Closteroviridae*. Similarities found between members of the genus *Closterovirus* and lettuce infectious yellows virus (LIYV, genus *Crinivirus*) [20] strongly indicate that the morphology and method of virion assembly are likely conserved within this family [21].

Grapevine leafroll-associated virus 3 (GLRaV-3) is arguably the most well-characterised member of the genus *Ampelovirus*. In contrast to the numerous functional studies performed on the proteins encoded by closterovirus ORFs, the functions of proteins encoded by ampeloviruses have largely been inferred by sequence homology to related viruses (Figure 1b) [22]. Moreover, with advances in the field of high throughput sequencing (HTS), research focus has shifted to sequencing-based strategies [23]. Limited functional studies have been conducted on grapevine virus encoded proteins, and the majority of current research focuses on identifying differentially expressed genes and elucidating the aetiology of grapevine diseases [24,25,26,27,28]. In this study, two complementary techniques were employed to identify interactions between proteins encoded by GLRaV-3 with the aim to gain a better understanding of the role of virus-encoded proteins in virion assembly and movement of members of the genus *Ampelovirus*.

## 2. Materials and Methods

### 2.1. Source Material

A *Vitis vinifera* cv. Cabernet Sauvignon own-rooted plant, maintained in a temperature-controlled greenhouse (Stellenbosch University, Matieland, South Africa) and singly infected with GLRaV-3 isolate GP18 (variant group II, EU259806) was used as a source of virus RNA. Phloem, leaf, and petiole material were pooled, and high-quality total RNA was extracted using a modified cetyltrimethylammonium bromide (CTAB) protocol [29]. To confirm the presence of GLRaV-3 in the sample, a diagnostic two-step RT-PCR was performed. cDNA was synthesized from 1 µg of total RNA primed with 150 ng random hexadeoxyribonucleotides (Promega, Madison, WI, USA) using Maxima Reverse Transcriptase and Thermo Scientific RiboLock RNase Inhibitor (ThermoFisher Scientific, Waltham, MA, USA) according to manufacturer’s instructions. A PCR reaction was then performed using KAPA Taq DNA Polymerase (Roche, Midrand, South Africa) according to manufacturer’s instructions, with primers targeted towards GLRaV-3 ORF 1a [30]. PCR reactions of 25 µL contained 2.5 µL cDNA 1X KAPA Taq buffer (Roche, Midrand, South Africa), 0.2 mM of each dNTP, forward and reverse primers at 0.4 µM each, and 1 U of KAPA Taq DNA Polymerase (Roche, Midrand, South Africa). PCRs were performed under the following conditions: 5 min initial denaturation at 95 °C; 35 cycles of denaturation (15 s at 95 °C), annealing (15 s at 53 °C), and extension (15 s at 72 °C); a final extension at 72 °C for 7 min. Amplicons were separated by electrophoresis and visualised on a 1% Agarose-TAE gel containing 0.5 µg/mL ethidium bromide.

### 2.2. Construction of Yeast Two-Hybrid Vectors

For the yeast two-hybrid (Y2H) analyses, yeast bait and prey plasmids pGBKT7-DB and pGADT7-AD (Clontech, Mountain View, CA, USA) were used. To amplify and clone GLRaV-3 ORFs into pGBKT7-DB and pGADT7-AD, primers targeted to each ORF with the addition of restriction enzyme (RE) sites were designed (Table S1). Using the cDNA described above, PCRs were performed using Phusion High-Fidelity DNA Polymerase (New England BioLabs, Ipswich, MA, USA). Each 50 µL PCR reaction contained 5 µL cDNA, 1X Phusion HF Buffer (New England Biolabs, Ipswich, MA, USA), 0.2 mM of each dNTP, forward and reverse primers at 0.5 µM each, DMSO at a final concentration of 3%, and 0.5 U of Phusion High-Fidelity DNA Polymerase (New England Biolabs, Ipswich, MA, USA). PCR conditions were as follows: 30 s initial denaturation at 98 °C; 35 cycles of denaturation (10 s at 98 °C), annealing (30 s at primer-specific T_m_), and extension (30 s/kb at 72 °C); with a final extension at 72 °C for 7 min. Amplicons of the correct size were excised and purified using a Zymoclean Gel DNA Recovery Kit (Zymo Research, Irvine, CA, USA). Purified amplicons and vector backbones were digested using the appropriate REs (Table S1) and ligated using T4 DNA ligase (New England BioLabs, Ipswich, MA, USA) according to the manufacturer’s instructions. Cloning of ORF 1a was performed in two segments due to its size. First, nt 1–1310 of ORF 1a were amplified using the *GLRaV-3 ORF 1a (5′)* primer set (Table S1) and cloned into pGADT7-AD and pGBKT7-DB using the methods described above to obtain pGADT7::1a-1 and pGADT7::1a-1. Then, nt 3006–6714 of ORF 1a were amplified using the *GLRaV-3 ORF 1a (3′)* primer set, after which the amplicon was excised and purified using a Zymoclean Gel DNA Recovery Kit (Zymo Research, Irvine, MA, USA). Both of the 1a-1 backbones and the purified amplicon were digested using BshTI and BamHI (Thermo Scientific, Waltham, MA, USA), purified using a Zymoclean Gel DNA Recovery Kit (Zymo Research, Irvine, CA, USA) and ligated using T4 DNA ligase (New England BioLabs, Ipswich, MA, USA) according to the manufacturer’s instructions. *Escherichia coli* strain JM109 was transformed with the ligated products. Colonies were screened using primers *Y2H Screen F* with *Y2H BR* (for bait plasmids) or *Y2H AR* (for prey plasmids) (Table S1), with the exception of those transformed with pGADT7::1a and pGBKT7::1a, which were screened using primers targeted towards GLRaV-3 ORF 1a [30]. Each colony was picked with a sterile toothpick and suspended in 10 µL PCR master mix containing 1X KAPA Taq buffer (Roche, Midrand, South Africa), 0.2 mM of each dNTP, forward and reverse primers at 0.4 µM each, and 0.5 U of KAPA Taq DNA Polymerase (Roche, Midrand, South Africa). PCRs using *Y2H Screen F* with *Y2H BR* or *Y2H AR* were performed under the following conditions: 5 min initial denaturation at 94 °C; 25 cycles of denaturation (30 s at 94 °C), annealing (30 s at 51 °C), and extension (1 min/kb at 72 °C); a final extension at 72 °C for 7 min. PCRs using the primers targeted towards ORF 1a were performed under the same conditions, with the exception of an increased annealing temperature of 53 °C. Colonies containing inserts of the correct size were inoculated in 5 mL LB supplemented with 50 µg/mL kanamycin and grown overnight at 37 °C, followed by a plasmid extraction using the GeneJET Plasmid Miniprep Kit (Thermo Scientific, Waltham, MA, USA). The recombinant plasmids were confirmed by Sanger sequencing using *Y2H Screen F*, *Y2H BR* (for bait plasmids), and *Y2H AR* (for prey plasmids) (Table S1). Because of the large insert size of plasmids containing ORF 1a, in addition to the sequencing reactions using the vector primers, RE digests were performed using SacI (pGADT7::1a) and BauI (pGBKT7::1a) (Thermo Scientific, Waltham, MA, USA) to confirm the correct inserts. Two additional sequencing reactions were performed using *p55 seq F* and *R* (Table S1) to confirm the sequences of pGADT7::p55 and pGBKT7::p55.

### 2.3. Yeast Transformation, Autoactivation and Toxicity Screening

Yeast cultures were grown on minimal, synthetically defined (SD) media consisting of a yeast nitrogen base without amino acids (Sigma-Aldrich, Johannesburg, South Africa), 2% (*w*/*v*) glucose, and the appropriate yeast synthetic drop-out medium supplement (Sigma-Aldrich): SD without tryptophan (SD/-Trp), SD without leucine (SD/-Leu), SD without tryptophane and leucine (DDO), or SD without histidine, tryptophane, leucine, and adenine (QDO). All yeast media were supplemented with 50 µg/mL kanamycin.

Strains, vectors, and inserts are referred to as: strain[vector::insert]. Using the Yeastmaker™ Yeast Transformation System 2 kit (Clontech, Mountain View, CA, USA) and following the manufacturer’s instructions, bait plasmids—pGBKT7::empty, a set of 13 pGBKT7 plasmids each containing one of the GLRaV-3 ORFs, and control plasmids pGBKT7-p53 and pGBKT7-Lam (Clontech, Mountain View, CA, USA)—were transformed into *Saccharomyces cerevisiae* strain Y2HGold and grown on SD/-Trp at 30 °C for five days. Similarly, prey plasmids—pGADT7::empty, a set of 13 pGADT7 plasmids each containing one of the GLRaV-3 ORFs, and control plasmid pGADT7-T (Clontech, Mountain View, CA, USA)—were transformed into *S. cerevisiae* strain Y187 and grown on SD/-Leu at 30 °C for five days. Successful transformation was confirmed by performing a colony PCR on transformed yeast using the Y2H screening primers (Table S1). Each yeast colony was picked with a sterile toothpick and resuspended in 10 µL 0.1 M NaOH. Resuspended cells were lysed at 37 °C for 10 min. PCR reactions were then performed using 2.5 µL of lysed cells as a template in 25 µL reactions containing 1X KAPA Taq buffer (Roche, Midrand, South Africa), 0.2 mM of each dNTP, forward and reverse primers at 0.4 µM each, and 1 U of KAPA Taq DNA Polymerase (Roche, Midrand, South Africa). PCRs were performed under the following conditions: 5 min initial denaturation at 95 °C; 35 cycles of denaturation (30 s at 95 °C), annealing (30 s at 51 °C), and extension (1 min/kb at 72 °C); and a final extension at 72 °C for 7 min.

To evaluate plasmids for toxicity, the growth of yeast containing each plasmid was compared to the same strain containing the empty version of that plasmid. Autoactivation was determined by performing a small-scale mating according to the Matchmaker® Gold Yeast Two-Hybrid System user Manual (PT4084-1, Takara Bio USA, 2010, p12) with each of the respective bait plasmids in Y2HGold with Y187[pGADT7::empty]. Mated yeast was plated on DDO containing 40 µg/mL X-α-Gal (Clontech, Mountain View, CA, USA) and 200 ng/mL Aureobasadin A (Clontech, Mountain View, CA, USA) (DDO/X/A) and QDO containing 40 µg/mL X-α-Gal and 200 ng/mL Aureobasadin A (QDO/X/A).

### 2.4. SDS-PAGE and Western Blotting

Yeast protein extractions were performed according to Kushnirov (2000) [31]. Extracted proteins were separated by 8% SDS-PAGE and blotted onto an Amersham™ Hybond^®^ LFP PVDF membrane (Merck, Johannesburg, South Africa). Bait protein expression was detected with the GAL4 DNA-BD Monoclonal Antibody, and prey protein expression with the GAL4 AD Monoclonal Antibody (Clontech, Mountain View, CA, USA), according to the manufacturer’s instructions. A Goat Anti-Mouse IgG (whole molecule)—Alkaline Phosphatase antibody (Sigma, Johannesburg, South Africa) was used as a secondary antibody and BCIP/NBT was used as the substrate (SigmaFast™ BCIP^®^/NBT tablets, Merck, Johannesburg, South Africa). Duplicate SDS-PAGE gels were subjected to Coomassie brilliant blue staining [32] to confirm successful protein extraction.

### 2.5. Y2H Small-Scale Matings and Screening for Protein Interactions

Small-scale matings were performed between each of the respective bait plasmids in Y2HGold and each respective prey plasmid in Y187 according to instructions in the Matchmaker^®^ Gold Yeast Two-Hybrid System User Manual (PT4084-1, Takara Bio USA, 2010, p12). Screening of the interaction of proteins that were toxic in Y187 was performed using co-transformation of Y2HGold with these plasmids and each of the respective GLRaV-3 pGBKT7 constructs, according to the Yeastmaker™ Yeast Transformation System 2 User Manual (PT1172-1, Clontech Laboratories USA, 2010, p7). Y187[pGADT7-T] was mated with Y2HGold[pGBKT-p53] and Y2HGold[pGBKT7-Lam] to serve as positive and negative controls, respectively. Mated and co-transformed yeast was plated on DDO selective media, and the presence of both plasmids was confirmed by a colony PCR using Y2H screening primers (Table S1), as described above. One colony from each respective mating or co-transformation was streaked onto DDO, DDOXA, and QDOXA plates and grown at 30 °C for 3–5 days to screen for interacting protein pairs.

### 2.6. Bimolecular Fluorescence Complementation Vectors

GLRaV-3 ORFs 2–7 and 10 were cloned into bimolecular fluorescence complementation (BiFC) binary vectors pSPYCE(M), pSPYCE(MR), pSPYNE173, and pSPYNE(R)173 [33] using primers provided in Table S1 and the same method described for the construction of the Y2H plasmids. To serve as an indicator of the success of infiltration, a red fluorescent protein-expressing plasmid pGPTVII-mCherry was constructed by cloning the mCherry gene from pmCherry (Clontech, Mountain View, CA, USA) into binary vector pGPTVII using primers and restriction enzymes provided in Table S1. *Arabidopsis thaliana* protein kinase CIPK24 was used as a negative control in all BiFC assays. Total RNA was extracted from *A. thaliana* using the Maxwell^®^ 16 LEV Plant RNA Kit (Promega, Madison, WI, USA). From this, the CIPK24 gene was amplified using the primers provided in Table S1, and cloned into each of the four BiFC vectors to obtain pSPYCE(M)::CIPK24, pSPYCE(MR)::CIPK24, pSPYNE173::CIPK24 and pSPYNE(R)173::CIPK24. All vectors were verified by Sanger sequencing. Also used in the BiFC assay was p19, a suppressor of gene silencing encoded by tomato bushy stunt virus cloned into the backbone pCAMBIA1300.

### 2.7. Bimolecular Fluorescence Complementation Assays in Nicotiana benthamiana

*Agrobacterium tumefaciens* strain GV3101/pMP90 was transformed with the respective plasmids using electroporation at 2.2 kV (MicroPulser Electroporator, Bio-Rad, Hercules, CA, USA). BiFC assays were performed according to Waadt and Kudla [34]. *Nicotiana benthamiana* plants were maintained in a growth room between 21 °C and 24 °C with a 16-h daylight regimen.

A summary of the BiFC workflow can be found in Supplementary Figure S1. For every protein pair screened, eight combinations of constructs containing the N- or C-terminal fragment of YFP fused to the N- or C-terminus of the proteins of interest, are possible [35]. Therefore, BiFC assays were conducted following a two-step approach. First, preliminary BiFC experiments were performed to identify the combination with the highest average efficacy of YFP reconstitution for each putative interaction. Following this, secondary BiFC experiments, this time with the inclusion of negative controls, were conducted only for protein pairs in which YFP reconstitution was prominent in at least one of the combinations screened during preliminary experiments. Two sets of BiFC assays were performed in this way. Validation assays were performed to confirm interactions found using the Y2H system. In addition to this, a set of BiFC screening assays was conducted to identify two-way interactions among GLRaV-3 proteins encoded by the quintuple gene block (p5, HSP70h, p55, CP, and CPm) and GLRaV-3 p20B.

In the preliminary experiments, three leaves on each of two 5–6-week-old *N. benthamiana* plants were infiltrated with *A. tumefaciens* GV3101/pMP90 containing p19 (OD600 = 0.3), pGPTVII-mCherry (OD600 = 0.5), and two complementary BiFC constructs, each encoding one of the proteins in the pair being screened (OD600 = 0.5). The undersides of infiltrated leaves were assayed for fluorescence 3 days post-infiltration. Images were captured using a Carl Zeiss ELYRA PS.1 LSM 780 confocal laser scanning microscope (Carl Zeiss, Jena, Germany) equipped with an EC Plan-Neofluar 10x/0.3 M27 objective, and an Andor iXon DU-885 EM-CCD camera run by the ZEN 2011 imaging software (Carl Zeiss, Jena, Germany). Water was used as an imaging medium. Excitation and emission wavelengths were 561 nm and 588–632 nm for mCherry, with laser power set to 2% (8 µW) and a detector gain of 600. YFP excitation and emission wavelengths were 488 nm and 517–552 nm, respectively. For all fusion protein combinations that did not include GLRaV-3 p20B, the power of the laser emitting a wavelength of 488 nm was set to 10% (106 µW) and the 517–552 nm detector gain was set to 900. However, an increase in the strength of the YFP signal in protein combinations involving p20B required the settings to be adjusted to avoid oversaturation. For imaging of these leaves, the power of the 488 nm laser was reduced to 5% (53 µW), whereas the 517–552 nm detector gain was reduced to 780. Images were processed using Fiji [36]. For every image, maximum intensity Z-projections were performed on the YFP and mCherry channels. Following that, the mean pixel intensity of each channel was calculated within the image. To quantify the efficacy of YFP reconstitution within each leaf, the mean intensity of mCherry was subtracted from the mean intensity of YFP ($MI_{YFP}-MI_{mCherry}=MI_{diff}$).

Secondary BiFC experiments comprised four different co-infiltrations, each also containing p19 and pGPTVII-mCherry:

1. The highest performing combination of orientations (Protein A and B)
2. Protein A with *A. thaliana* CIPK24 in the orientation of Protein B
3. Protein B with *A. thaliana* CIPK24 in the orientation of Protein A
4. No YFP constructs, to serve as an internal reference for expression strength and a baseline of background fluorescence.

Each co-infiltration was performed on three leaves on each of three *N. benthamiana* plants. The respective *MI_diff_* values of the nine leaves from each set of infiltrated plants were calculated. A Shapiro-Wilk test for normality was performed on the data using SciPy version 1.4.1 [37], followed by an ANOVA and a Tukey’s honestly significantly differenced test using Statsmodels version 0.11.0 [38] to identify samples that differed significantly from the controls. *p*-values smaller than 0.01 were assumed to be significant.

### 2.8. GLRaV-3 Transmembrane Protein Analyses

To compare the predicted membrane topology of GLRaV-3 p5 to that of the transmembrane proteins encoded by other members of the family *Closteroviridae*, the amino acid sequences of the transmembrane movement proteins of BYV and CTV, along with those of GLRaV-3 and other members of the genus *Ampelovirus*, were subjected to in silico membrane protein topology and signal peptide prediction using TOPCONS [39]. A complete list of GenBank accession numbers of these proteins is provided in Table S3.

## 3. Results

### 3.1. Yeast Two-Hybrid Assays

A complete set of bait (pGBKT7-DB) and prey (pGADT7-AD) vectors, each containing one of the 13 GLRaV-3 ORFs, was constructed and transformed into *S. cerevisiae* Y2HGold (bait) and Y187 (prey). Although no bait plasmids proved toxic in Y2HGold, Y187 containing pGADT7::p5, pGADT7::p21, and pGADT7::p20B, respectively, showed delayed or no growth on SD/-Leu agar plates. Screening of prey plasmids encoding these proteins against the complete set of bait plasmids was therefore conducted by co-transformation of Y2HGold. Furthermore, in Y2HGold[pGBKT7::p21] mated with Y187[pGADT7::empty], all four of the reporter genes were autoactivated. Consequently, it was omitted from further screenings.

Expression of fusion proteins was determined by performing a western blot on yeast protein extracts. The expected sizes of the fusion proteins encoded by pGBKT7::Methyltransferase–helicase and pGADT7::Methyltransferase–helicase were 265.94 kDa and 263.68 kDa, respectively. However, western blots revealed that the fusion proteins encoded by these vectors were between 52 and 76 kDa in size. This is in accordance with the estimated size of these proteins following autocatalytic cleavage of the leader protease domains, the position of which has been predicted by a multiple amino acid sequence alignment of other members of the family *Closteroviridae* [40]. Fusion proteins expressed by Y2HGold containing pGBKT7::RdRP and pGBKT7::p55 were smaller than expected, while no expression of fusion proteins could be detected in Y187 containing pGADT7::RdRP and pGADT7::p6. Consequently, these plasmids were left out of subsequent Y2H screening experiments.

In a set of one-on-one, small-scale mating experiments, Y2HGold cultures containing each of the bait plasmids were mated with Y187 cultures containing each of the prey plasmids, with the exception of pGADT7::p5, pGADT7::p21, and pGADT7::p20B, which were screened using co-transformation of Y2HGold. Only four of the mated cultures exhibited growth and a blue colour on QDO/X/A media, indicating an interaction between the two proteins (Table 1):

- Y2HGold[pGBKT7::HSP70h] x Y187[pGADT7::HSP70h]
- Y2HGold[pGBKT7::CP] x Y187[pGADT7::HSP70h]
- Y2HGold[pGBKT7::CP] x Y187[pGADT7::p6]
- Y2HGold[pGBKT7::p20B] x Y187[pGADT7::p20B]

### 3.2. Bimolecular Fluorescence Complementation Assays

**Figure 2:**
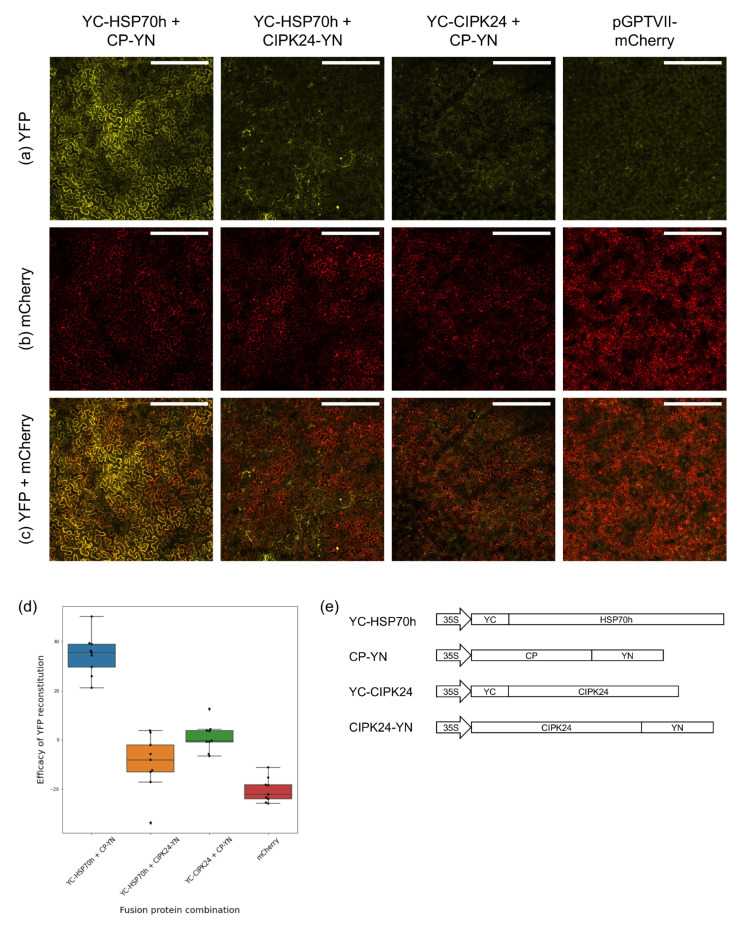
Summary of the BiFC assay conducted to investigate the interaction of GLRaV-3 heat shock protein 70 homolog (HSP70h) and GLRaV-3 major coat protein (CP) *in planta*. Fluorescent microscopy images are of the underside of *Nicotiana benthamiana* leaves infiltrated with various combinations of bimolecular fluorescence complementation (BiFC) constructs indicated above each image. All combinations of BiFC constructs were co-infiltrated with silencing suppressor p19, and pGPTVII-mCherry, expressing red fluorescent protein mCherry. The red colour represents mCherry emissions, while the yellow represents the signal emitted by reconstituted YFP. Scale bars indicate a distance of 500 microns. (**a**) YFP emission for each combination; (**b**) mCherry emission for each combination; (**c**) A composite image of both YFP and mCherry emission; (**d**) A box-and-whiskers plot summarising the efficacy of YFP reconstitution of each protein combination, as quantified by subtracting the mean intensity of mCherry from the mean intensity of YFP. Individual plot points are shown as black dots; (**e**) Schematic presentation of the expression cassettes for YC or YN fusion proteins. YC-HSP70h: C-terminal fragment of yellow fluorescent protein (YFP) fused to N-terminus of HSP70h; CP-YN: N-terminal fragment of YFP fused to C-terminus of CP; YC-CIPK24: C-terminal fragment of yellow fluorescent protein (YFP) fused to N-terminus of *Arabidopsis thaliana* protein kinase CIPK24 (CIPK24); CIPK24-YN: N-terminal fragment of YFP fused to C-terminus of CIPK24.

The results of the BiFC assays in which the *MI_diff_* of the GLRaV-3 protein combinations were significantly higher than those of controls are summarised in Table 1. Three of the four protein-protein interactions found in yeast were confirmed in *N. benthamiana*: HSP70h-HSP70h, p20B-p20B, and HSP70h-CP (Figure 2, Figures S3 and S6, Table S2). No YFP signal was detectable in leaves infiltrated with BiFC plasmids encoding YFP fragments fused to the CP and p6 (Table 1). Five additional protein-protein interactions not detected in the Y2H experiments were revealed in the second set of BiFC assays. Self-interaction of CP and p5 was observed (Figures S2 and S5), while HSP70h, CP, and CPm all interacted with p20B (Figures S7–S9). It is worth noting that the YFP signal in protein combinations that included p20B was higher than those of other combinations, necessitating a change in imaging conditions for that channel in those combinations and their associated controls to avoid overexposure.

### 3.3. GLRaV-3 Transmembrane Protein Analyses

**Figure 3:**
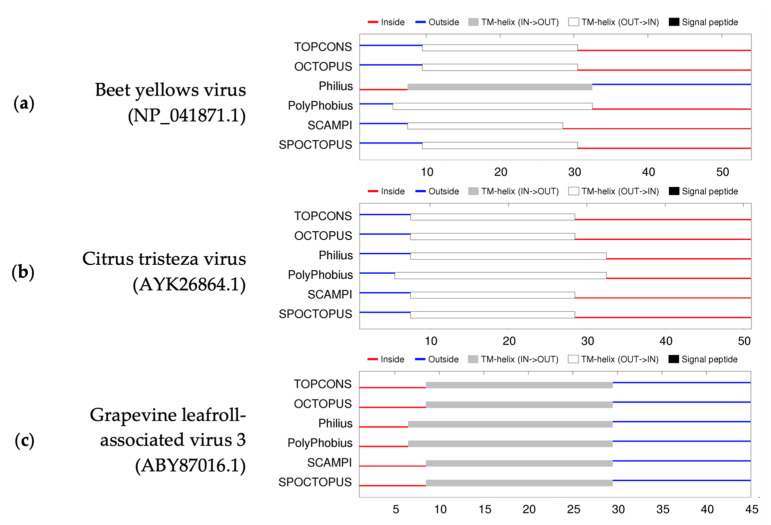
A visual summary of the membrane topology predictions of (**a**) beet yellows virus (BYV) p6, (**b**) citrus tristeza virus (CTV) p6, and (**c**) grapevine leafroll-associated virus 3 (GLRaV-3) p5 using six different algorithms in TOPCONS [39]. Amino acid positions are indicated below each figure. Blue lines represent amino acids predicted to reside on the outside lumen of ER; while red lines indicate amino acids predicted to be localised inside the cytosol. Areas predicted as transmembrane helices (TM) are illustrated in grey or white. No signal peptides were detected.

A visual summary of the predictions of the membrane helices and topology of the movement proteins of BYV, CTV, and GLRaV-3 by six different prediction methods using TOPCONS is shown in Figure 3. The consensus membrane topology prediction of BYV p6 is consistent with what was previously reported and validated experimentally: the hydrophobic N-terminus protrudes into the ER lumen, while the hydrophilic C-terminus faces the cytosol [13,41]. While the predicted topology of CTV p6 follows the same pattern, the membrane topology of GLRaV-3 seems to be reversed. A summary of the TOPCONS results of BYV, CTV, GLRaV-3 isolates representing different variants, and other members of the genus *Ampelovirus* can be found in Table S3. No signal peptides were detected in any of the sequences.

## 4. Discussion

The functions of proteins encoded by GLRaV-3 ORFs have largely been inferred by sequence homology to related viruses from the genus *Closterovirus*, most notably beet yellows virus (BYV) and citrus tristeza virus (CTV). In this study, two independent protein–protein interaction assays were conducted on proteins encoded by GLRaV-3 ORFs in order to gain insight on the function of these ORFs by comparison to what has been reported for other members of the family *Closteroviridae*.

Closteroviruses encode a conserved QGB encoding structural and movement proteins involved in virion assembly and movement [42]. The p5 protein is the first protein encoded by the GLRaV-3 QGB and is homologous to BYV p6, a small hydrophobic transmembrane protein targeted towards the endoplasmic reticulum (ER) [13]. BYV p6 is essential, but not sufficient, for cell-to-cell movement of the virus [19]. Functional studies performed on the p6 encoded by CTV showed similar results [43]. Evidence of the self-interaction of GLRaV-3 p5 was obtained in the BiFC assay (Table 1, Figure S2), although it was undetectable using the Y2H system. Because integral membrane proteins are retained at cellular membranes, interactions of these proteins are not readily detectable using a standard Y2H assay and requires a modified approach [44]. No interaction was detected between GLRaV-3 p5 and any of the other proteins screened in either of the assays. It was established that CTV p6 is not involved in virion formation [43]. Although the exact mechanism by which p6 facilitates cell-to-cell movement is not known, mutation analysis showed that both the C- and N-termini of this protein are necessary for BYV translocation [13]. As both assays used in this study have limitations, failure to detect a protein interaction using these assays does not provide definitive proof of its absence.

Formation of disulfide bonds that lead to the dimerisation of the BYV transmembrane movement protein relies on two key factors. First, the presence of a cysteine residue near the N-terminus of the protein [13]. Second, the localisation of the N-terminal end of the protein in the ER lumen [45]. GLRaV-3 transmembrane proteins do not, however, satisfy either of these conditions. There is no cysteine residue near the N-terminal of GLRaV-3 p5. Examination of the amino acid sequences of this protein of other ampeloviruses indicated that this is the case for all members of the genus. Several sequence motifs implicated in the dimerisation of transmembrane proteins have been identified [46]. One such motif, Thr-X-X-X-Thr, with X representing any amino acid, occurs at the C-terminus of the majority of p5 proteins encoded by GLRaV-3 isolates (Table S3) and could present a possible alternative for dimerisation. However, these motifs are abundant in transmembrane proteins and the dimerization of a protein cannot be predicted solely on the presence of such motifs [46]. Furthermore, the N-terminus of GLRaV-3 p5 is predicted to face the cytosol, while the C-terminus is predicted to be located in the ER lumen. This differs from those of the closteroviruses and the other members of the genus *Ampelovirus*, apart from blackberry vein banding associated virus (AGS48180.1) and grapevine leafroll-associated virus 13 (BAU80810.1), for which a transmembrane region could not be reliably detected using all algorithms (Table S3). No signal peptides were detected in the sequences of any of the transmembrane proteins, indicating that they are most likely type II membrane-bound proteins instead of type I [47]. Further research is required to confirm the membrane topology of GLRaV-3 p5 in living cells and to identify residues essential for the dimerisation of ampelovirus movement proteins.

Although many viruses utilise cellular HSP70 chaperones to assist in replication or virion assembly [48], to date, viruses in the family *Closteroviridae* are the only known viruses to encode their own [49]. HSP70 chaperones share a distinct structural organisation, with a conserved N-terminus followed by an ATPase domain [50] and a more variable C-terminus containing a peptide-binding pocket [51]. The high level of similarity between closteroviral and cellular HSP70s allows for the extrapolation of possible functions and mechanisms of actions of virus-encoded HSP70hs based on knowledge about cellular molecular chaperones [19]. Initially, cellular HSP70s were theorised to act as monomers. However, it has since been revealed that both bacterial and eukaryotic HSP70 family members are able to oligomerise [52,53]. The results of both the Y2H and BiFC assays revealed self-interaction of the GLRaV-3 HSP70h. The presence of ~10 molecules of HSP70h in a single beet yellows virus particle could suggest the formation of multimers by this protein [54], however, the ability of plant virus-encoded HSP70h to form multimeric structures has not been reported prior to this study.

ORF5 of GLRaV-3 produces p55, a member of a family of ~60 kDa proteins encoded by members of the family *Closteroviridae* [55]. Proteins in this family consist of a highly conserved C-terminus embedded into the virion, and a more variable N-terminal domain that is exposed on the virion surface [8,19]. Mutational analyses of p64, the homologous protein encoded by BYV, determined that this protein is essential, but not sufficient, for intercellular translocation of the virus [19]. Failure of the Y2H assay to detect any interactions with GLRaV-3 p55 could be due to the attachment of the DNA binding domain, or transcriptional activation domain, fused to the C-terminus of p55, rendering it inaccessible for interaction. However, no interactions were detected using BiFC, either.

It has been reported that the homologs of both p55 and HSP70h are integral components of the virion head of BYV [54,56]. Although the association of p55 homologs and HSP70h with the virion head consisting of CPm is established, neither of the assays in this study detected interactions between these three proteins. A prior study revealed that inactivation of BYV p64 prevented incorporation of HSP70h into the virion head, and vice versa [56]. Furthermore, inactivation of CPm reduced incorporation of HSP70h and p64 into the virion head of BYV [56]. It has been shown that BYV p64 possesses RNA-binding properties [9] and that both HSP70h and p61, the CTV homolog of BYV p64, are required to restrict CPm encapsidation to the 5′ terminal end of the genome [57]. Together, these results could indicate a multi-way interaction between these three proteins, possibly also requiring the presence of virus RNA. Therefore, the possibility of an interaction between p55, HSP70h, and CPm cannot be excluded.

The flexuous, filamentous virions of members of the family *Closteroviridae* are mainly composed of multiple subunits of two distinct coat proteins [49]. The CP encapsidates the majority of the viral genome, whereas the CPm is the main component of the virion head and covers the 5′ ~650 nucleotides of viral RNA [10]. Neither of these proteins were shown to self-interact in the Y2H assay. These results are in accordance with Y2H assays conducted on the ampelovirus, pineapple mealybug wilt-associated virus 2 (PMWaV-2) [58]. However, self-interaction of the GLRaV-3 CP was detected using the BiFC assay. Previous studies concluded that the CP is capable of encapsidating genomic RNA without requiring CPm, p64, or HSP70h in BYV [7]. The same was not true for CTV, however, the authors theorised that the failure to detect intact, full-length virions could have been due to the instability of virions without the presence of HSP70h [10]. Neither of the assays detected self-interaction of the CPm. It has been shown that both the HSP70h and the homologs of p55 are required for proper formation of virion head structures [7,43,54,56]. Previously, it was speculated that CP formation is necessary to provide a structural platform for attachment of the virion head [7]. More recent studies have revealed that, in the absence of a functional CP, p55 homolog, and HSP70h, the CPm of BYV and CTV are capable of encapsidating the complete RNA genome of the respective viruses, starting at the 5′ end of the genome [10,13]. This, however, requires the presence of two stem-and-loop structures within the 5′ NTR of the genome [59]. Failure to detect a self-interaction of CPm using either of the two assays employed in this study could indicate that another factor, possibly genomic RNA, is necessary to initiate assembly of GLRaV-3 CPm. Additionally, no interaction between CP and CPm was found. As both HSP70h and p61 of CTV are required to restrict CPm encapsidation to the 5′ ~650 nucleotides of the genome, it has been suggested that these proteins might form a collar region between the head and body of virions [8] and that the CP and CPm do not physically interact.

A surprising interaction revealed by both assays was one between HSP70h and CP. As previously stated, HSP70h is not required for assembly of the virion body [7]. However, inactivation of HSP70h results in a prevalence of incomplete BYV virions and no detectable full size CTV virions [7,10]. HSP70h might play a role in virion stability by assisting in the formation and attachment of virion heads, as head disassembly may promote uncoating of viral RNA [7]. The protein–protein interaction between HSP70h and CP demonstrated in this study could suggest that HSP70h stabilises full-size virions by physical interaction with the CP. Closteroviral-encoded HSP70hs are associated with the plasmodesmata [60]. The proposed mechanism by which HSP70h mediates intercellular translocation of virions is based on a similar mode of action regulated by ATP hydrolysis employed by all cellular HSP70s. This involves repeated binding and releasing of target proteins to pull them through plasmodesmata [61,62,63]. Such a mechanism might also require physical binding of HSP70h to CP.

Although the results of the Y2H assay indicated an interaction between the CP and an unknown protein product of ORF2, no significant interaction could be observed between these proteins using BiFC. As ORF2 is not present in all GLRaV-3 variants [64] and its expression has not been proven [65], this seems unlikely to be a true interaction.

Perhaps the most unexpected set of interactions were those involving p20B, a protein that exhibited silencing suppressor activity in *N. benthamiana* [18]. The 3′ proximal ORFs of members of the family *Closteroviridae* are not well conserved among genera and do not share significant sequence or structural similarity [66]. However, similarly located ORFs in members of this family encode proteins involved in long-distance virus transport and suppression of host RNA defence response [14,15]. BYV silencing suppressor p21 forms octameric ring structures with an RNA binding inner surface [67], while the p20 protein of CTV exhibited strong self-interaction [68]. Similarly, PMWaV-2 p20, a local and systemic silencing suppressor, and p21, a systemic silencing suppressor, were both shown to form homodimers [58]. GLRaV-3 p20B showed self-interaction in both assays. It is therefore likely that the silencing mechanism of p20B also requires polymerisation. Due to the silencing suppression activity of this protein, the level of YFP fluorescence in plants infiltrated with any combination that included p20B was much higher than for any of the other plasmids, requiring an adjustment of microscopy settings. The *MI_diff_* values marked with an asterisk in Table 1 should therefore not be directly compared to those of other protein pairs. The interactions of p20B with other GLRaV-3-encoded proteins share some similarities to those observed in other members of the family. BiFC assays indicated that, in addition to self-interaction, the GLRaV-3 p20B interacts with three of the four structural proteins of GLRaV-3: the HSP70h, CP, and CPm. BYV p20 was not identified as a silencing suppressor [15] but is indispensable for long-distance transport of the virus [12]. Although the exact mechanism by which p20 facilitates translocation of BYV is not known, mutational studies combined with atomic force microscopy strongly suggested that this protein forms the tip segment of the virion head structure [12], likely via interaction with HSP70h [14]. Interaction of the GLRaV-3 p20B with HSP70h suggests a similar morphology of the GLRaV-3 virion head. Whether the interaction of p20B with the two coat proteins is a result of its role in silencing suppression, long-distance transport, or both remains unclear. In this study, the interaction of an ampelovirus silencing suppressor with structural proteins HSP70h, CP, and CPm is reported. Further investigation of these interactions could lead to the elucidation of the functional mechanisms of virus-encoded silencing suppressors, and possibly indicates that this protein plays additional roles in the replication and spread of GLRaV-3.

This paper describes a comprehensive investigation on the protein–protein interactions of a member of the genus *Ampelovirus*. These results provide avenues for future research into the molecular mechanisms behind the assembly and spread of members of the family *Closteroviridae*.

## Figures and Tables

**Table 1:** Summary of grapevine leafroll-associated virus 3 protein–protein interactions identified using bimolecular fluorescence complementation (BiFC) and/or yeast two-hybrid (Y2H) assays.

Protein A	Protein B	Y2H Detection	Optimal Combination of BiFC Plasmid Orientations	Average *MI_diff_*
HSP70h	HSP70h	Yes	YC-HSP70h + YN-HSP70h	51.78
HSP70h	CP	Yes	YC-HSP70h + CP-YN	34.63
p20B	p20B	Yes	YC-p20B + YN-p20B	92.7 *
CP	p6	Yes	No YFP reconstitution observed	N/A
CP	CP	No	CP-YC + YN-CP	26.99
p5	p5	No	YC-p5 + YN-p5	113.86
HSP70h	p20B	No	YC-p20B + YN-HSP70h	45.55 *
CP	p20B	No	YC-p20B + YN-CP	55.66 *
CPm	p20B	No	YC-p20B + YN-CPm	27.24 *

YC: C-terminus of yellow fluorescent protein (YFP); YN: N-terminus of YFP. Location of protein of interest (POI) name relative to YC or YN indicates the orientation of the fusion protein: YC-POI: YC fused to N-terminus of POI; POI-YC: YC fused to C-terminus of POI, etc. HSP70h: heat shock protein 70 homolog; CP: coat protein; p20B: silencing suppressor p20B; p5: transmembrane movement protein p5; CPm: minor coat protein. The average efficacy of YFP reconstitution for each protein pair is quantified by *MI_diff_*: the mean intensity value of mCherry subtracted from the mean intensity value of YFP. * Due to stronger YFP signals of protein combinations that included silencing suppressor p20B, visualisation settings had to be adapted to avoid oversaturation in this channel.

## Data Availability

The data presented in this study are available in Supplementary Materials Figures S2–S9 and Tables S2 and S3.

## Supplementary Materials

The following supporting information can be downloaded at: https://www.mdpi.com/article/10.3390/v15010208/s1, Table S1: Summary of primers and restriction enzymes used to construct vectors and screen colonies; Table S2: Statistical analyses of BiFC data.; Figure S1: Graphical illustration of BiFC workflow; Figures S2–S9: Collection of images representing BiFC results and constructs for all protein combinations; Table S3: Predicted membrane topologies of citrus tristeza virus, beet yellows virus, isolates of grapevine leafroll-associated virus 3, and other members of the genus *Ampelovirus*.
