# Supplementary material for: Identification of Interactions between Proteins Encoded by Grapevine Leafroll-Associated Virus 3
Source: Viruses. 2023 Jan 11;15(1):208. doi: 10.3390/v15010208 (PMC9865355; doi:10.3390/v15010208)
Supplement: Supplementary file 1 [file viruses-15-00208-s001.zip › Figure S1.pdf]

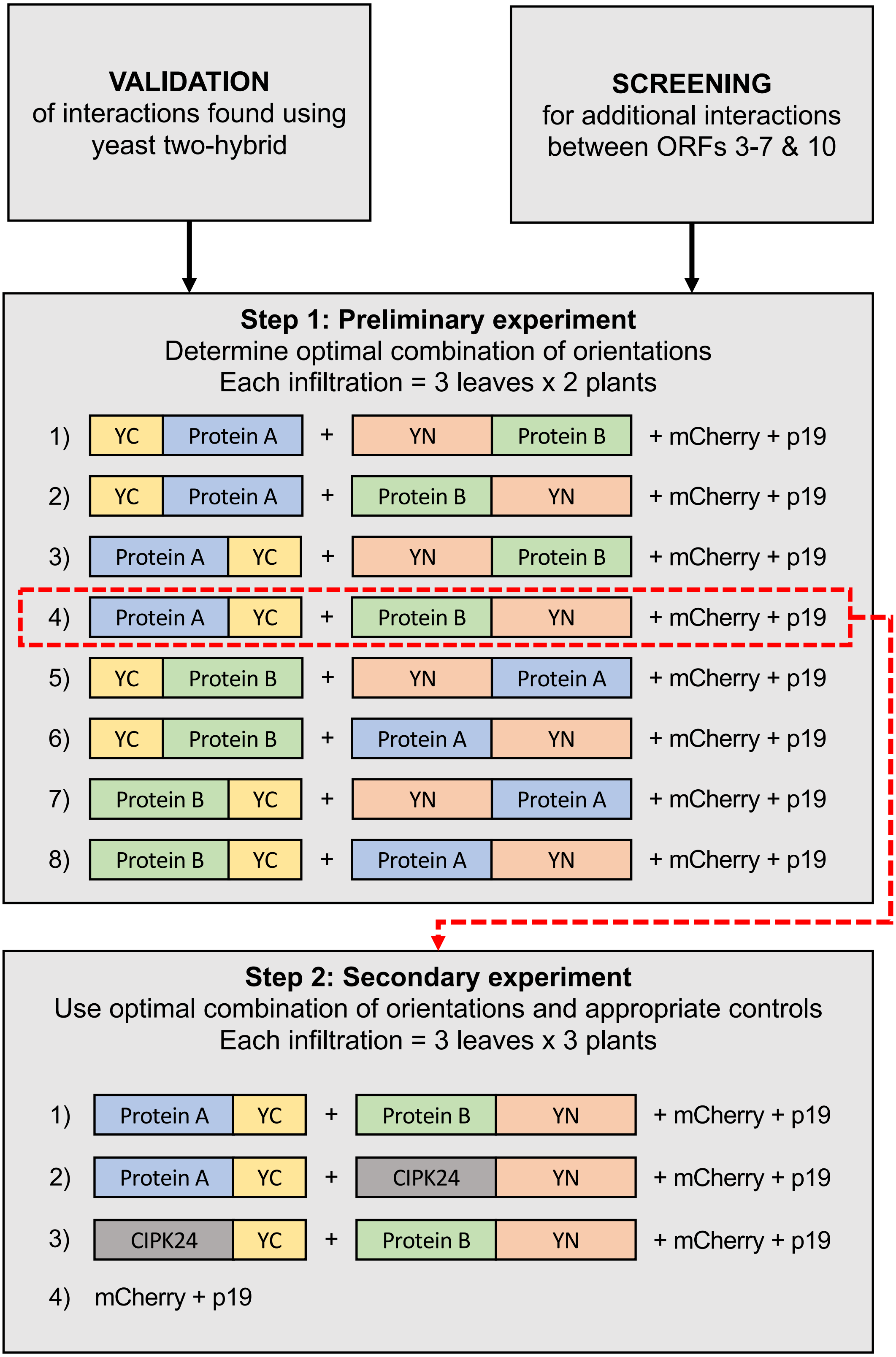

Figure S1: A summary of the bimolecular complementation workflow followed to perform validation and screening assays. mCherry: pGPTVII-mCherry, expressing red fluorescent protein mCherry; p19: silencing suppressor; YC: C-terminus of yellow fluorescent protein (YFP), YN: N-terminus of YFP.
