# Supplementary material for: Identification of Interactions between Proteins Encoded by Grapevine Leafroll-Associated Virus 3
Source: Viruses. 2023 Jan 11;15(1):208. doi: 10.3390/v15010208 (PMC9865355; doi:10.3390/v15010208)
Supplement: Supplementary file 1 [file viruses-15-00208-s001.zip › Figures S2-S9.pdf]

p5 – p5

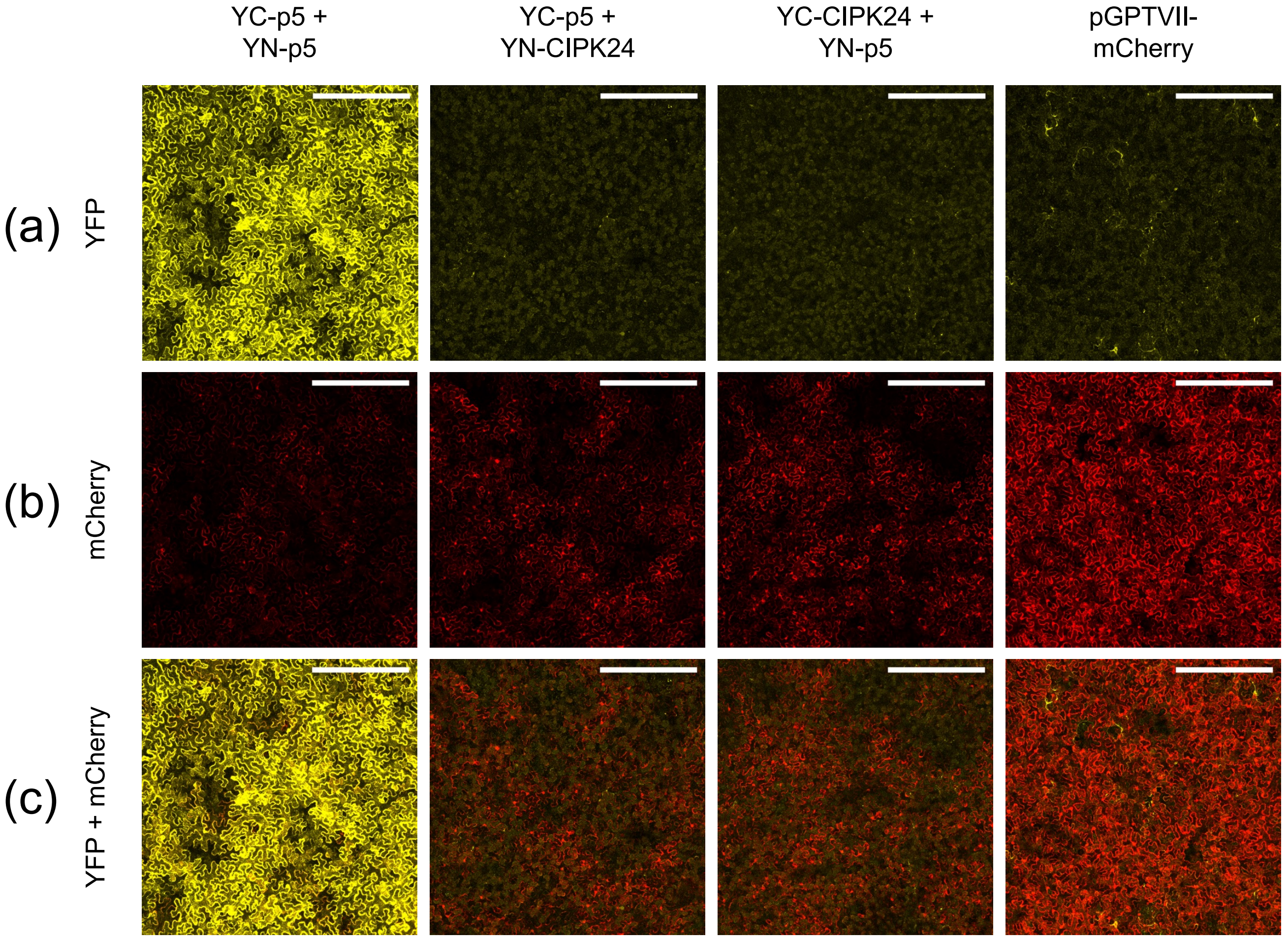

(d)

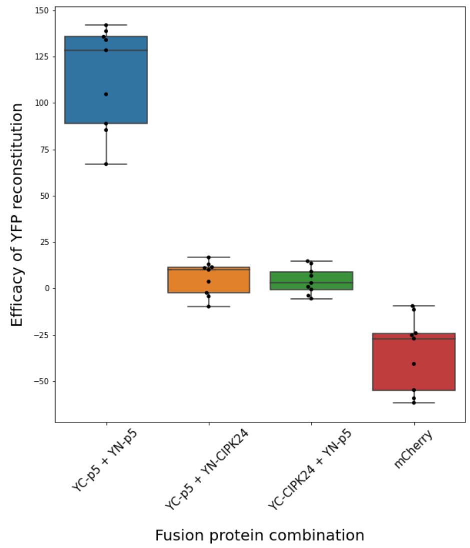

(e)

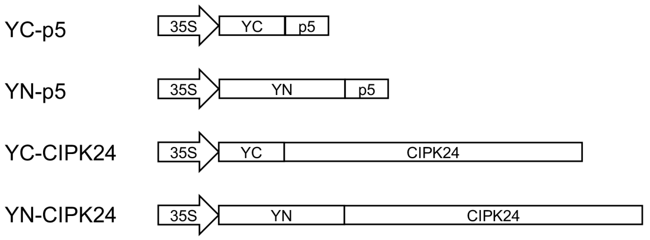

Figure S2: Summary of the BiFC assay conducted to investigate the self-interaction of GLRaV-3 p5 *in planta.* Fluorescent microscopy images are of the underside of *Nicotiana benthamiana* leaves infiltrated with various combinations of bimolecular fluorescence complementation (BiFC) constructs indicated above each image. All combinations of BiFC constructs were co-infiltrated with silencing suppressor p19, and pGPTVII-mCherry, expressing red fluorescent protein mCherry. The red colour represents mCherry emissions, while the yellow represents the signal emitted by reconstituted YFP. Scale bars indicate a distance of 500 microns. (**a**) The YFP emission for each combination; (**b**) The mCherry emission for each combination; (**c**) A composite image of both the YFP and mCherry emissions; (**d**) A box-and-whiskers plot summarising the efficacy of YFP reconstitution of each protein combination, as quantified by subtracting the mean intensity of mCherry from the mean intensity of YFP. Individual plot points are shown as black dots; (**e**) Schematic presentation of the expression cassettes for YC or YN fusion proteins. YC-p5: C-terminal fragment of yellow fluorescent protein (YFP) fused to N-terminus of p5; YN-p5 N-terminal fragment of YFP fused to N-terminus of p5; YC-CIPK24: C-terminal fragment of yellow fluorescent protein (YFP) fused to N-terminus of *Arabidopsis thaliana* protein kinase CIPK24 (CIPK24); YN-CIPK24: N-terminal fragment of YFP fused to N-terminus of CIPK24.

HSP70h – HSP70h

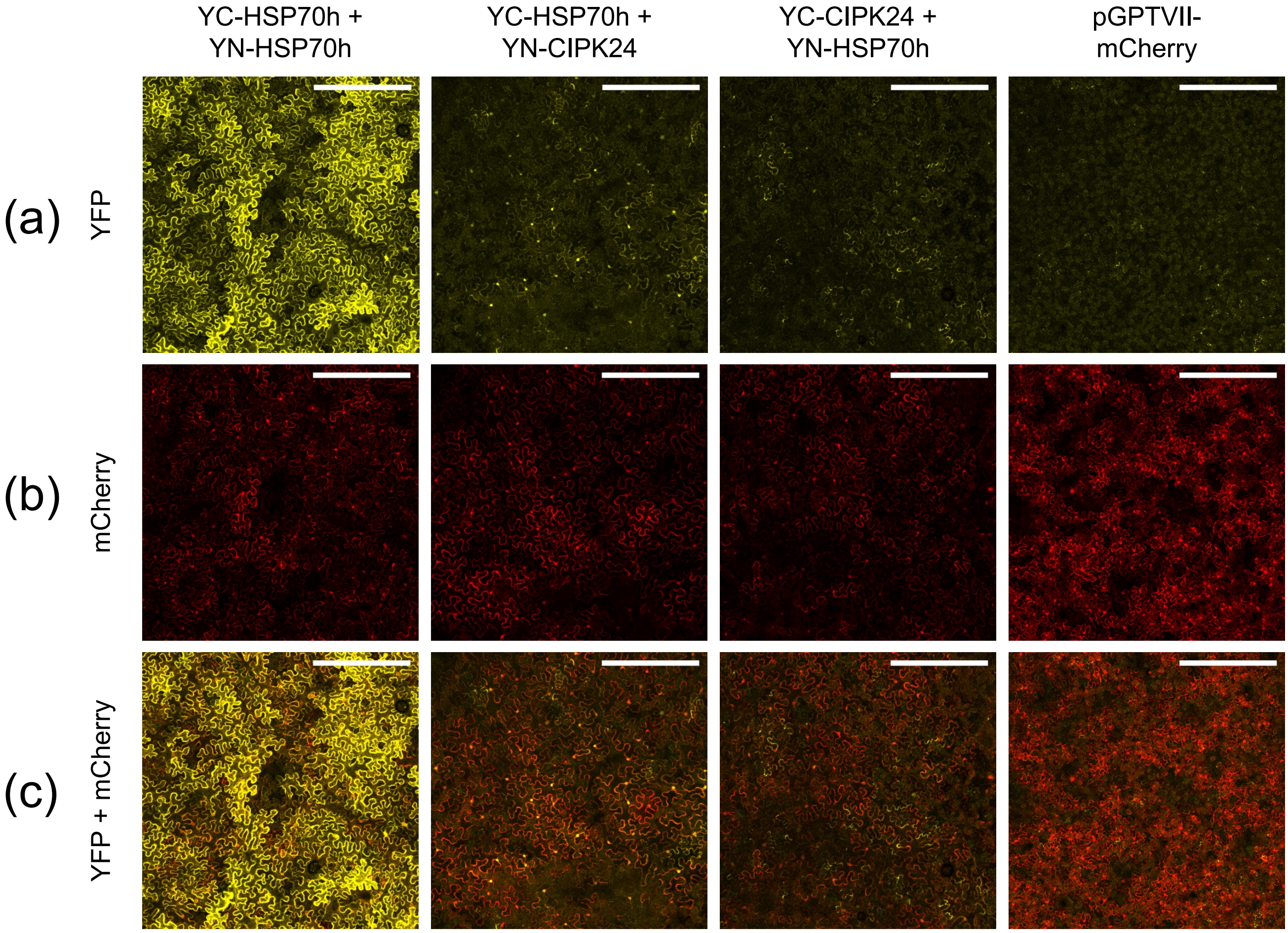

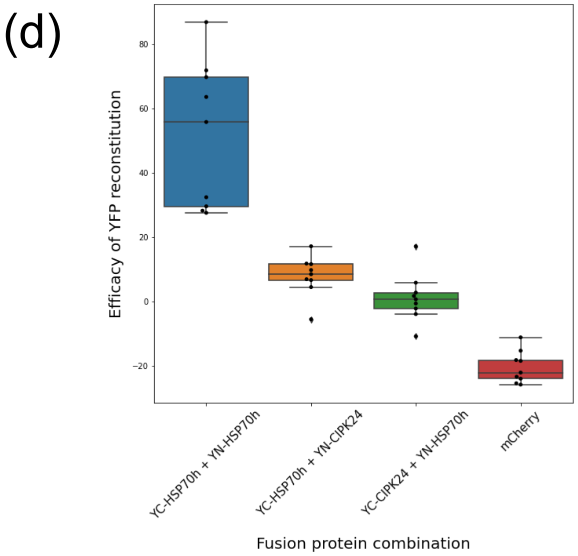

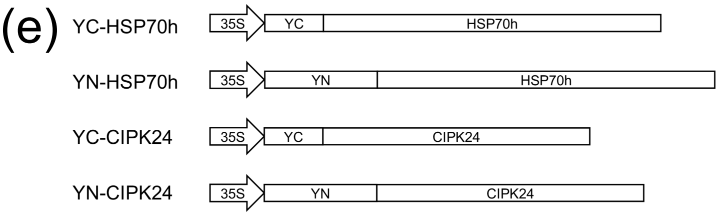

Figure S3: Summary of the BiFC assay conducted to investigate the self-interaction of GLRaV-3 heat shock protein 70 homolog (HSP70h) *in planta*. Fluorescent microscopy images are of the underside of *Nicotiana benthamiana* leaves infiltrated with various combinations of bimolecular fluorescence complementation (BiFC) constructs indicated above each image. All combinations of BiFC constructs were co-infiltrated with silencing suppressor p19, and pGPTVII-mCherry, expressing red fluorescent protein mCherry. The red colour represents mCherry emissions, while the yellow represents the signal emitted by reconstituted YFP. Scale bars indicate a distance of 500 microns. (**a**) The YFP emission for each combination; (**b**) The mCherry emission for each combination; (**c**) A composite image of both the YFP and mCherry emissions; (**d**) A box-and-whiskers plot summarising the efficacy of YFP reconstitution of each protein combination, as quantified by subtracting the mean intensity of mCherry from the mean intensity of YFP. Individual plot points are shown as black dots; (**e**) Schematic presentation of the expression cassettes for YC or YN fusion proteins. YC-HSP70h: C-terminal fragment of yellow fluorescent protein (YFP) fused to N-terminus of HSP70h; YN-HSP70h: N-terminal fragment of YFP fused to N-terminus of HSP70h; YC-CIPK24: C-terminal fragment of yellow fluorescent protein (YFP) fused to N-terminus of *Arabidopsis thaliana* protein kinase CIPK24 (CIPK24); YN-CIPK24: N-terminal fragment of YFP fused to N-terminus of CIPK24.

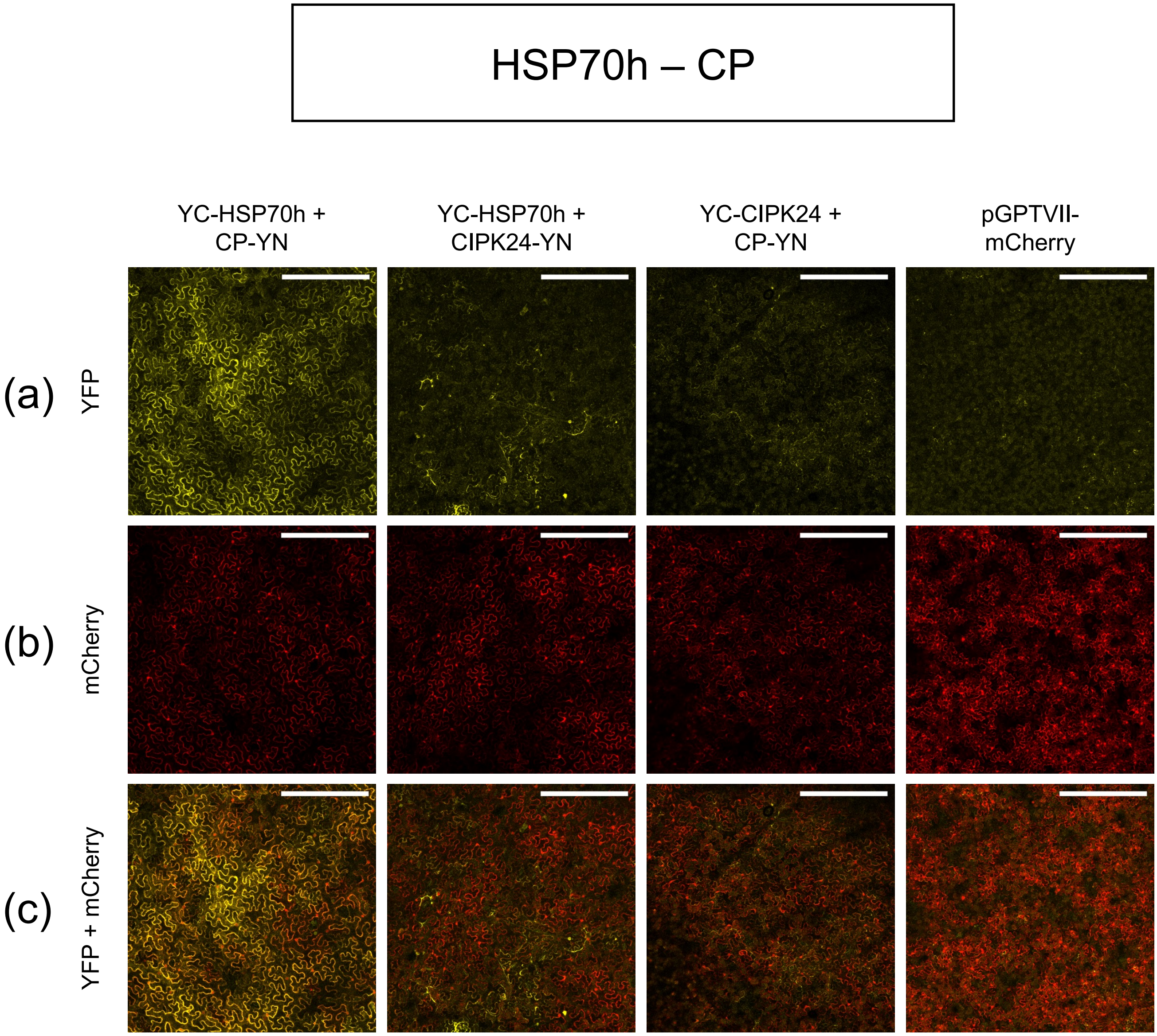

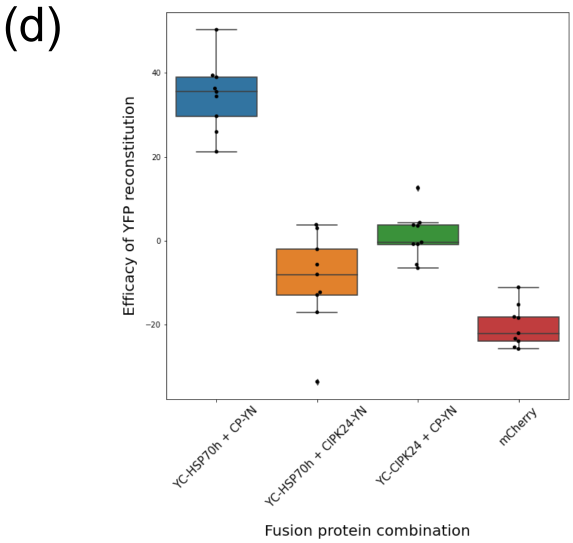

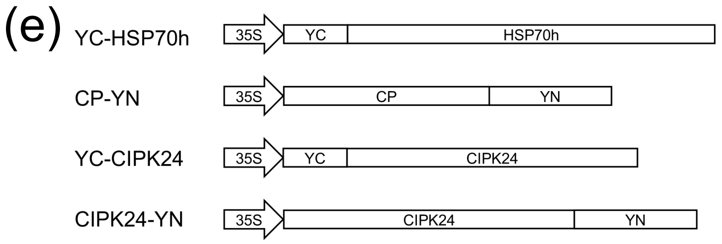

Figure S4: Summary of the BiFC assay conducted to investigate the interaction of GLRaV-3 heat shock protein 70 homolog (HSP70h) and GLRaV-3 major coat protein (CP) *in planta*. Fluorescent microscopy images are of the underside of *Nicotiana benthamiana* leaves infiltrated with various combinations of bimolecular fluorescence complementation (BiFC) constructs indicated above each image. All combinations of BiFC constructs were co-infiltrated with silencing suppressor p19, and pGPTVII-mCherry, expressing red fluorescent protein mCherry. The red colour represents mCherry emissions, while the yellow represents the signal emitted by reconstituted YFP. Scale bars indicate a distance of 500 microns. (**a**) The YFP emission for each combination; (**b**) The mCherry emission for each combination; (**c**) A composite image of both the YFP and mCherry emissions; (**d**) A box-and-whiskers plot summarising the efficacy of YFP reconstitution of each protein combination, as quantified by subtracting the mean intensity of mCherry from the mean intensity of YFP. Individual plot points are shown as black dots; (**e**) Schematic presentation of the expression cassettes for YC or YN fusion proteins. YC-HSP70h: C-terminal fragment of yellow fluorescent protein (YFP) fused to N-terminus of HSP70h; CP-YN: N-terminal fragment of YFP fused to C-terminus of CP; YC-CIPK24: C-terminal fragment of yellow fluorescent protein (YFP) fused to N-terminus of *Arabidopsis thaliana* protein kinase CIPK24 (CIPK24); CP-YN: N-terminal fragment of YFP fused to C-terminus of CIPK24.

CP-CP

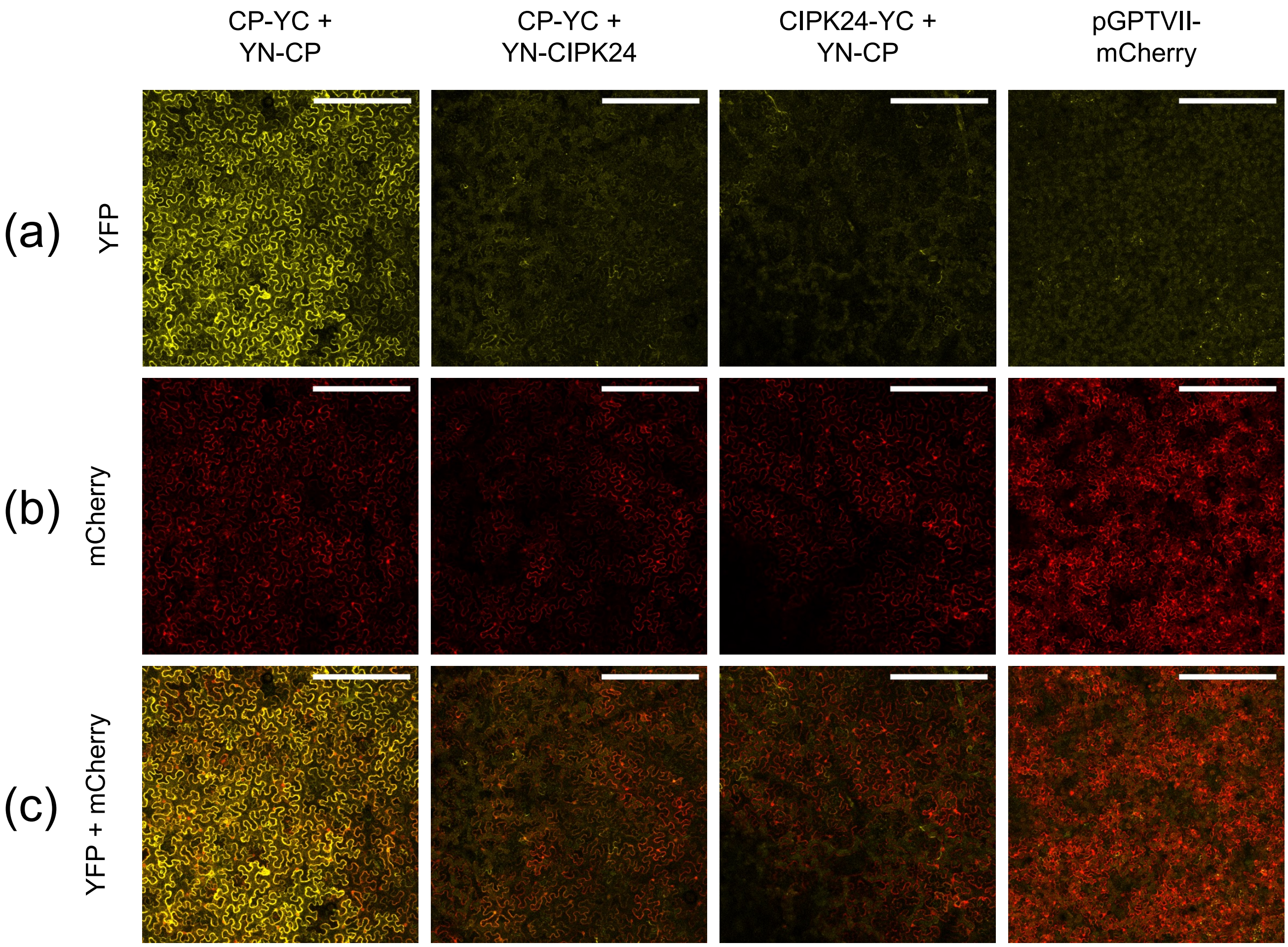

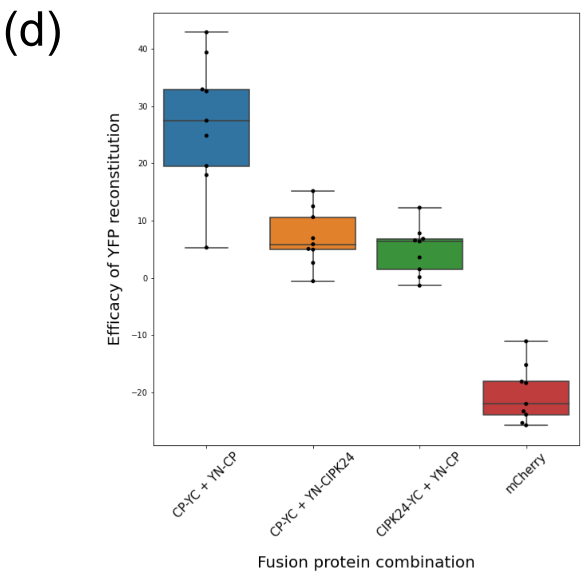

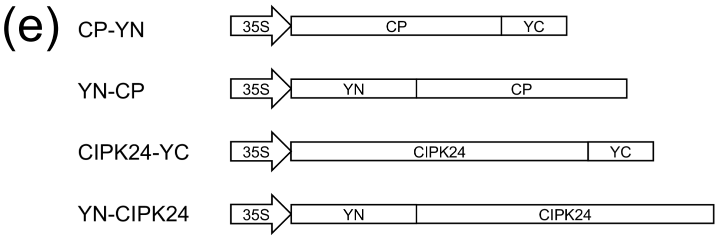

Figure S5: Summary of the BiFC assay conducted to investigate the self-interaction of GLRaV-3 major coat protein (CP) *in planta*. Fluorescent microscopy images are of the underside of *Nicotiana benthamiana* leaves infiltrated with various combinations of bimolecular fluorescence complementation (BiFC) constructs indicated above each image. All combinations of BiFC constructs were co-infiltrated with silencing suppressor p19, and pGPTVII-mCherry, expressing red fluorescent protein mCherry. The red colour represents mCherry emissions, while the yellow represents the signal emitted by reconstituted YFP. Scale bars indicate a distance of 500 microns. (**a**) The YFP emission for each combination; (**b**) The mCherry emission for each combination; (**c**) A composite image of both the YFP and mCherry emissions; (**d**) A box-and-whiskers plot summarising the efficacy of YFP reconstitution of each protein combination, as quantified by subtracting the mean intensity of mCherry from the mean intensity of YFP. Individual plot points are shown as black dots; (**e**) Schematic presentation of the expression cassettes for YC or YN fusion proteins. CP-YC: C-terminal fragment of yellow fluorescent protein (YFP) fused to C-terminus of CP; YN-CP: N-terminal fragment of YFP fused to N-terminus of CP; YC-CIPK24: C-terminal fragment of yellow fluorescent protein (YFP) fused to N-terminus of *Arabidopsis thaliana* protein kinase CIPK24 (CIPK24); YN-CIPK24: N-terminal fragment of YFP fused to N-terminus of CIPK24.

p20B – p20B

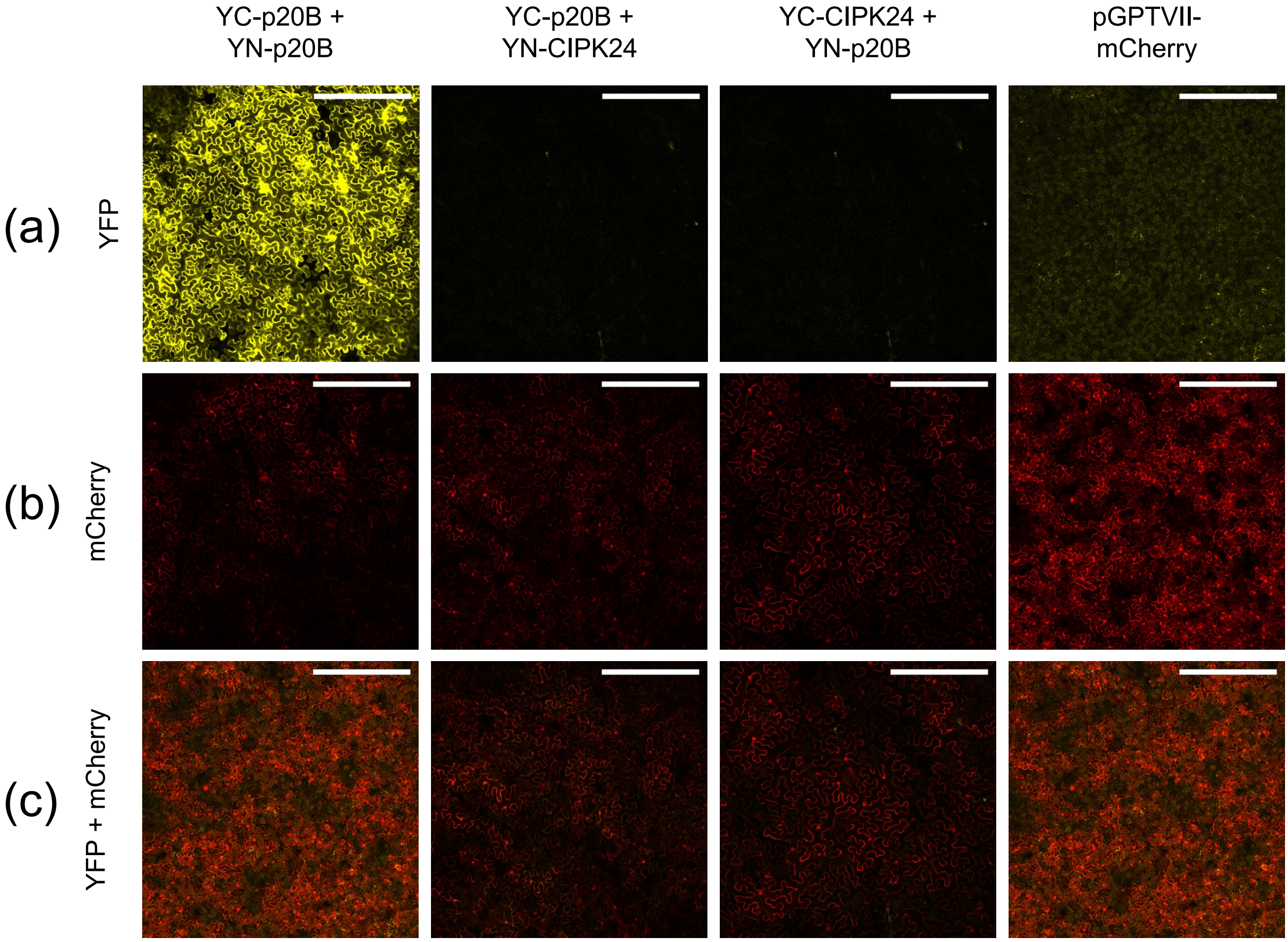

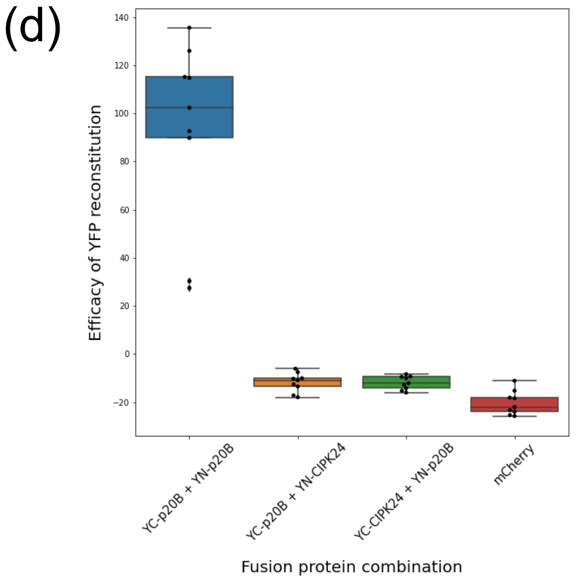

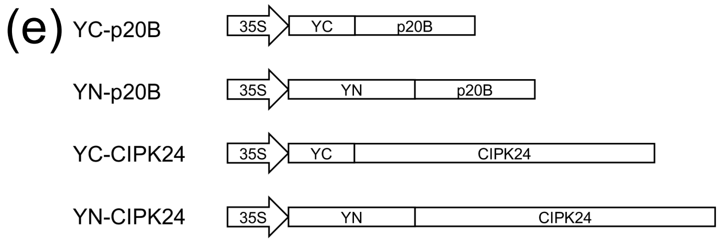

Figure S6: Summary of the BiFC assay conducted to investigate the self-interaction of GLRaV-3 p20B *in planta*. Fluorescent microscopy images are of the underside of *Nicotiana benthamiana* leaves infiltrated with various combinations of bimolecular fluorescence complementation (BiFC) constructs indicated above each image. All combinations of BiFC constructs were co-infiltrated with silencing suppressor p19, and pGPTVII-mCherry, expressing red fluorescent protein mCherry. The red colour represents mCherry emissions, while the yellow represents the signal emitted by reconstituted YFP. Scale bars indicate a distance of 500 microns. (**a**) The YFP emission for each combination; (**b**) The mCherry emission for each combination; (**c**) A composite image of both the YFP and mCherry emissions; (**d**) A box-and-whiskers plot summarising the efficacy of YFP reconstitution of each protein combination, as quantified by subtracting the mean intensity of mCherry from the mean intensity of YFP. Individual plot points are shown as black dots; (**e**) Schematic presentation of the expression cassettes for YC or YN fusion proteins. YC-p20B: C-terminal fragment of yellow fluorescent protein (YFP) fused to N-terminus of p20B; YN-p20B: N-terminal fragment of YFP fused to N-terminus of p20B; YC-CIPK24: C-terminal fragment of yellow fluorescent protein (YFP) fused to N-terminus of *Arabidopsis thaliana* protein kinase CIPK24 (CIPK24); YN-CIPK24: N-terminal fragment of YFP fused to N-terminus of CIPK24.

HSP70h – p20B

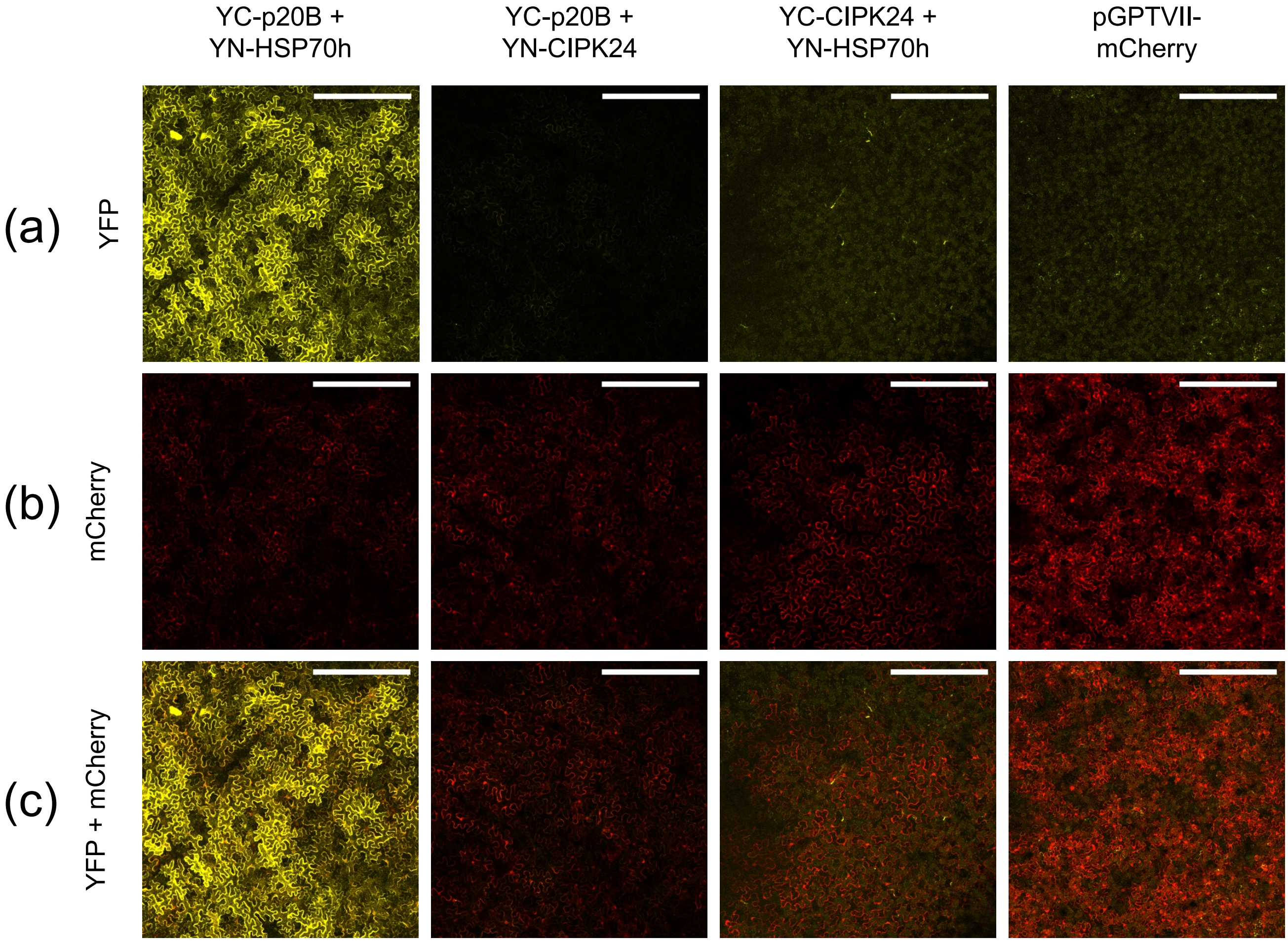

(d)

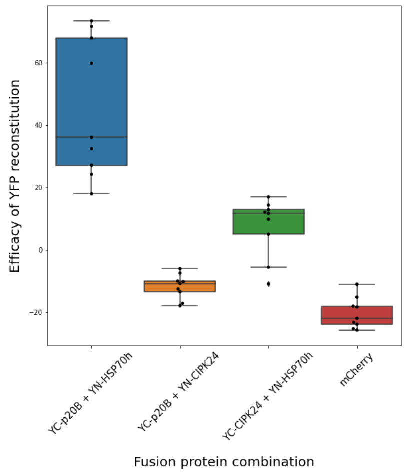

(e)

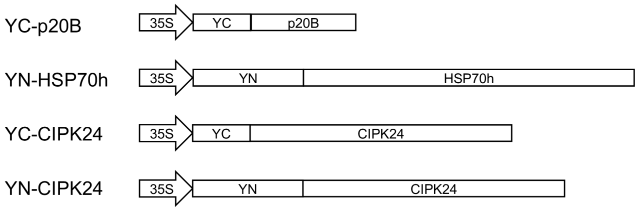

Figure S7: Summary of the BiFC assay conducted to investigate the interaction of GLRaV-3 heat shock protein 70 homolog (HSP70h) with GLRaV-3 p20B *in planta*. Fluorescent microscopy images are of the underside of *Nicotiana benthamiana* leaves infiltrated with various combinations of bimolecular fluorescence complementation (BiFC) constructs indicated above each image. All combinations of BiFC constructs were co-infiltrated with silencing suppressor p19, and pGPTVII-mCherry, expressing red fluorescent protein mCherry. The red colour represents mCherry emissions, while the yellow represents the signal emitted by reconstituted YFP. Scale bars indicate a distance of 500 microns. (**a**) The YFP emission for each combination; (**b**) The mCherry emission for each combination; (**c**) A composite image of both the YFP and mCherry emissions; (**d**) A box-and-whiskers plot summarising the efficacy of YFP reconstitution of each protein combination, as quantified by subtracting the mean intensity of mCherry from the mean intensity of YFP. Individual plot points are shown as black dots; (**e**) Schematic presentation of the expression cassettes for YC or YN fusion proteins. YC-HSP70h: C-terminal fragment of yellow fluorescent protein (YFP) fused to N-terminus of HSP70h; YN-p20B: N-terminal fragment of YFP fused to N-terminus of p20B; YC-CIPK24: C-terminal fragment of yellow fluorescent protein (YFP) fused to N-terminus of *Arabidopsis thaliana* protein kinase CIPK24 (CIPK24); YN-CIPK24: N-terminal fragment of YFP fused to N-terminus of CIPK24.

CP – p20B

(a) YFP

(b) mCherry

(c) YFP + mCherry

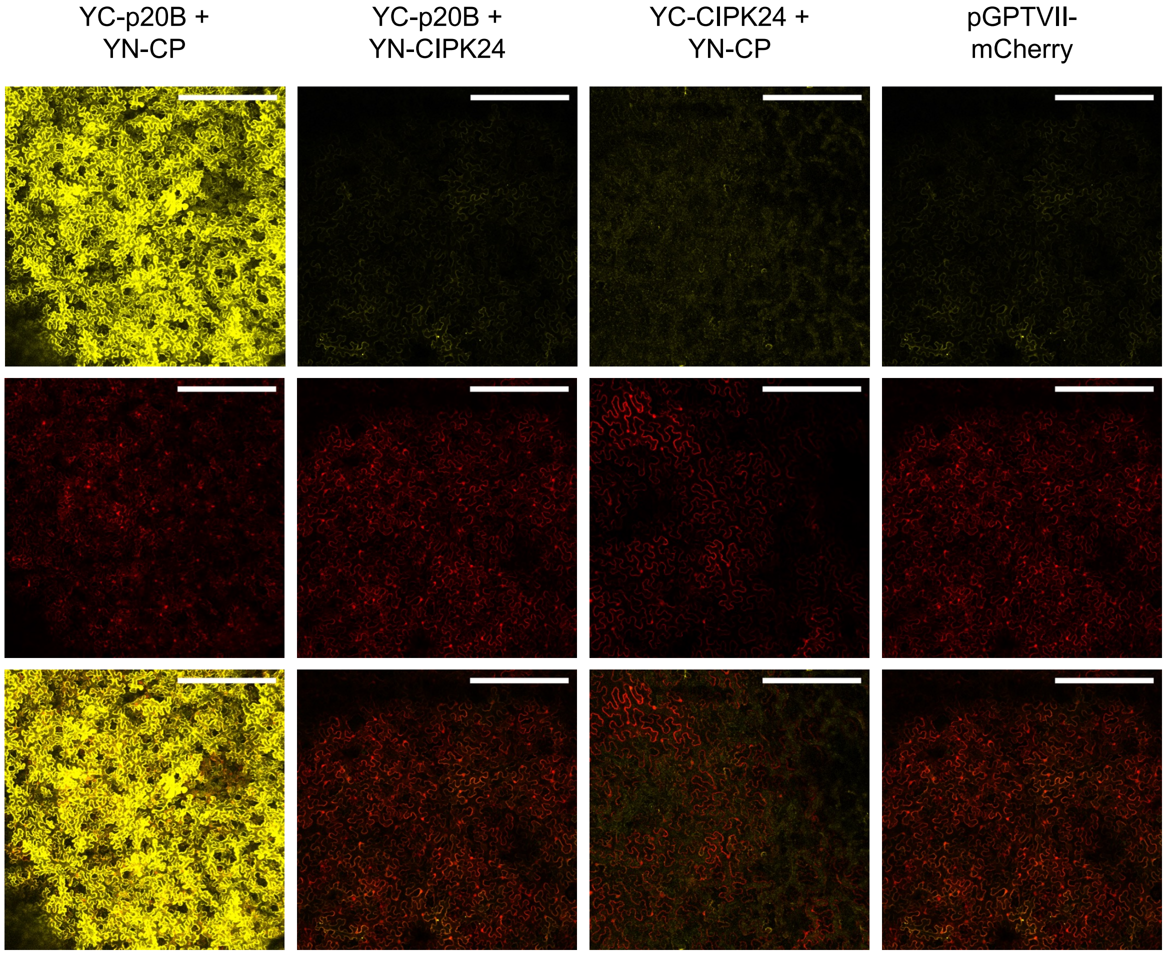

(d)

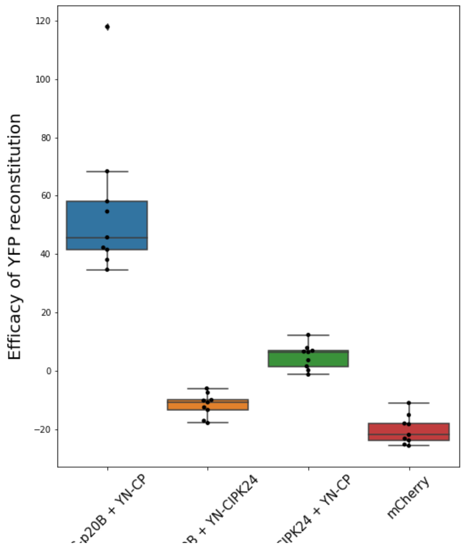

(e)

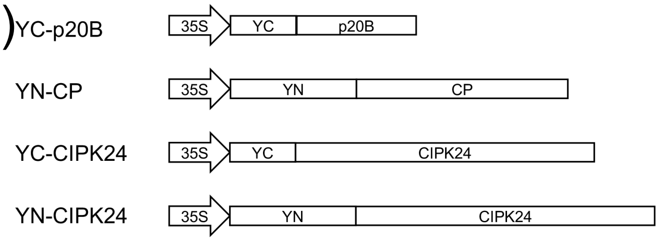

Figure S8: Summary of the BiFC assay conducted to investigate the interaction of GLRaV-3 major coat protein (CP) with GLRaV-3 p20B *in planta*. Fluorescent microscopy images are of the underside of *Nicotiana benthamiana* leaves infiltrated with various combinations of bimolecular fluorescence complementation (BiFC) constructs indicated above each image. All combinations of BiFC constructs were co-infiltrated with silencing suppressor p19, and pGPTVII-mCherry, expressing red fluorescent protein mCherry. The red colour represents mCherry emissions, while the yellow represents the signal emitted by reconstituted YFP. Scale bars indicate a distance of 500 microns. (**a**) The YFP emission for each combination; (**b**) The mCherry emission for each combination; (**c**) A composite image of both the YFP and mCherry emissions; (**d**) A box-and-whiskers plot summarising the efficacy of YFP reconstitution of each protein combination, as quantified by subtracting the mean intensity of mCherry from the mean intensity of YFP. Individual plot points are shown as black dots; (**e**) Schematic presentation of the expression cassettes for YC or YN fusion proteins. YC-CP: C-terminal fragment of yellow fluorescent protein (YFP) fused to N-terminus of CP; YN-p20B: N-terminal fragment of YFP fused to N-terminus of p20B; YC-CIPK24: C-terminal fragment of yellow fluorescent protein (YFP) fused to N-terminus of *Arabidopsis thaliana* protein kinase CIPK24 (CIPK24); YN-CIPK24: N-terminal fragment of YFP fused to N-terminus of CIPK24.

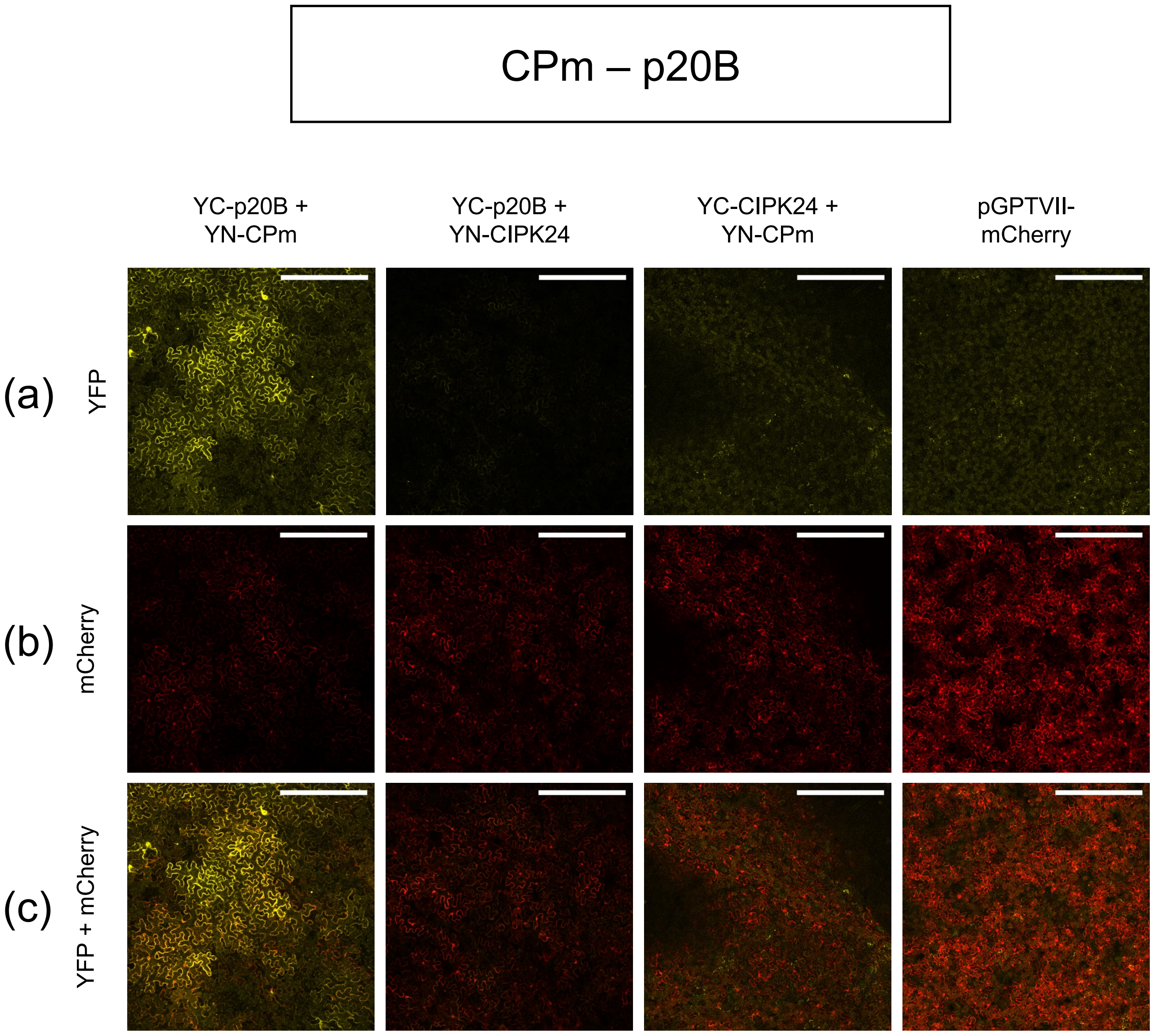

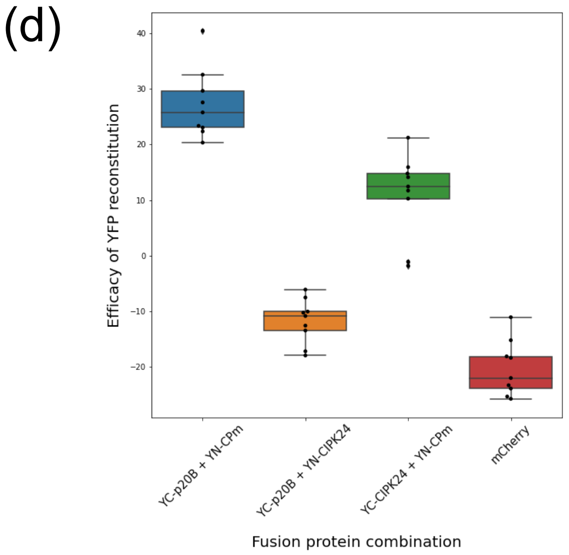

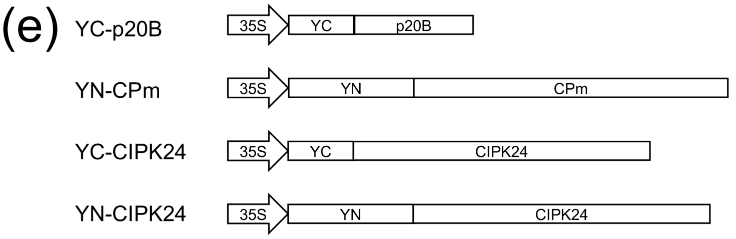

Figure S9: Summary of the BiFC assay conducted to investigate the interaction of GLRaV-3 minor coat protein (CPm) with GLRaV-3 p20B *in planta*. Fluorescent microscopy images are of the underside of *Nicotiana benthamiana* leaves infiltrated with various combinations of bimolecular fluorescence complementation (BiFC) constructs indicated above each image. All combinations of BiFC constructs were co-infiltrated with silencing suppressor p19, and pGPTVII-mCherry, expressing red fluorescent protein mCherry. The red colour represents mCherry emissions, while the yellow represents the signal emitted by reconstituted YFP. Scale bars indicate a distance of 500 microns. (**a**) The YFP emission for each combination; (**b**) The mCherry emission for each combination; (**c**) A composite image of both the YFP and mCherry emissions; (**d**) A box-and-whiskers plot summarising the efficacy of YFP reconstitution of each protein combination, as quantified by subtracting the mean intensity of mCherry from the mean intensity of YFP. Individual plot points are shown as black dots; (**e**) Schematic presentation of the expression cassettes for YC or YN fusion proteins. YC-CPm: C-terminal fragment of yellow fluorescent protein (YFP) fused to N-terminus of CPm; YN-p20B: N-terminal fragment of YFP fused to N-terminus of p20B; YC-CIPK24: C-terminal fragment of yellow fluorescent protein (YFP) fused to N-terminus of *Arabidopsis thaliana* protein kinase CIPK24 (CIPK24); YN-CIPK24: N-terminal fragment of YFP fused to N-terminus of CIPK24.
